# Intracellular Application of an Asparaginyl Endopeptidase for Producing Recombinant Head-to-Tail Cyclic Proteins

**DOI:** 10.1021/jacsau.3c00591

**Published:** 2023-11-20

**Authors:** T. M. Simon Tang, Jody M. Mason

**Affiliations:** † Department of Life Sciences, University of Bath, Claverton Down, Bath, North Somerset BA2 7AY, U.K.

**Keywords:** cyclic peptides, peptide ligation, asparaginyl endopeptidases, OaAEP1, ribosomally synthesized and post-translationally modified peptides, RiPPs

## Abstract

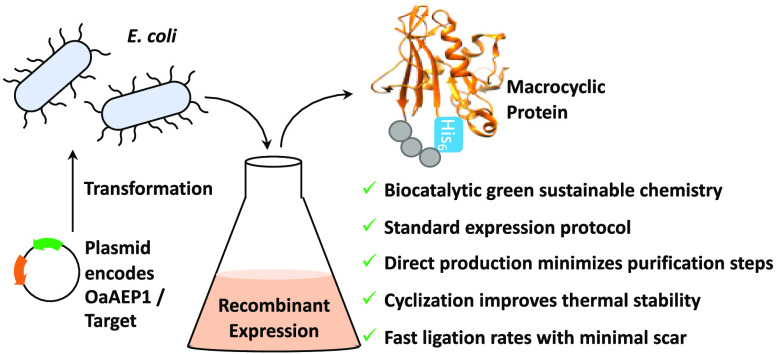

Peptide backbone cyclization is commonly observed in nature and is increasingly applied to proteins and peptides to improve thermal and chemical stability and resistance to proteolytic enzymes and enhance biological activity. However, chemical synthesis of head-to-tail cyclic peptides and proteins is challenging, is often low yielding, and employs toxic and unsustainable reagents. Plant derived asparaginyl endopeptidases such as OaAEP1 have been employed to catalyze the head-to-tail cyclization of peptides *in vitro*, offering a safer and more sustainable alternative to chemical methods. However, while asparaginyl endopeptidases have been used *in vitro* and in native and transgenic plant species, they have never been used to generate recombinant cyclic proteins in live recombinant organisms outside of plants. Using dihydrofolate reductase as a proof of concept, we show that a truncated OaAEP1 variant C247A is functional in the *Escherichia coli* physiological environment and can therefore be coexpressed with a substrate protein to enable concomitant *in situ* cyclization. The bacterial system is ideal for cyclic protein production owing to the fast growth rate, durability, ease of use, and low cost. This streamlines cyclic protein production via a biocatalytic process with fast kinetics and minimal ligation scarring, while negating the need to purify the enzyme, substrate, and reaction mixtures individually. The resulting cyclic protein was characterized *in vitro*, demonstrating enhanced thermal stability compared to the corresponding linear protein without impacting enzyme activity. We anticipate this convenient method for generating cyclic peptides will have broad utility in a range of biochemical and chemical applications.

## Introduction

Ligase type asparaginyl endopeptidases (AEPs) such as butelase 1 and OaAEP1 have been employed to facilitate peptide ligation and head-to-tail (H2T) cyclization reactions *in vitro*.^1−14^ OaAEP1 facilitates H2T cyclization of peptides during the biosynthesis of cyclotides via an Asn-containing recognition sequence in the acidic vacuole of the plant species *Oldenlandia affinis*.^1^ Peptide ligation by the engineered OaAEP1-C247A variant proceeds with impressive catalytic efficiency via a short recognition sequence,^7,8^ offering application in site-specific protein modification to study post-translational modification (PTM) in cell biology research,^9^ production of protein–drug conjugates,^10^ and protein semisynthesis.^11,12^ Recombinant OaAEP1-C247A is commonly prepared as an inactive zymogen which can only be activated in acidic conditions (pH 3.6–4.0) postpurification.^8−12^ To simplify recombinant preparation and improve usability of OaAEP1-C247A, studies have been performed to identify truncated yet functional constructs which bypass the acid activation step.^7^ While OaAEP1-C247A exhibits optimal activity in acidic conditions (pH 5.0), the enzyme has been shown to remain active over a broad pH range (pH 4.5–7.4).^7^ The ability to bypass the larger zymogen, which can only be activated in acidic conditions found in the plant vacuole, and recombinantly produce the catalytic domain of OaAEP1, which has been shown to be active at physiological pH, opens many new avenues of research toward applications in live bacteria.

While methods for *in vitro* protein H2T cyclization using AEPs such as butelase 1 and OaAEP1 have been reported,^1−14^ purification of the enzyme and substrate and subsequent isolation of the cyclic product is time-consuming, and inconvenience becomes a major barrier for application. For example, recombinant OaAEP1 preparation requires two to four purification steps and an overnight activation step to yield only 1.8–2.0 mg of active enzyme from a one liter *E. coli* culture.^1,7,8,14,15^ The ability to introduce PTM within a host organism during recombinant expression offers a significant advantage over *in vitro* approaches, as it negates the need to purify individual protein components. Moreover, it offers a green and cost-effective method to produce high-value modified peptides and proteins^16^ and novel applications such as *in situ* modification during intracellular screening for peptide drug discovery.^17^

Cyclization is a powerful modification with applications across protein and peptide research.^18^ In peptide drug discovery, cyclization is often employed to improve drug-like properties as constraining the peptide into the bioactive conformation can enhance target binding affinity.^17,19−21^ Moreover, H2T cyclization removes cleavable N- and C-termini, which improves peptide stability against exopeptidases,^22,23^ and can assist in conferring cell penetrance owing to the removal of the amino- and carboxy-terminal charges.^24,25^ Protein cyclization can also improve thermal stability,^26,27^ a highly desirable attribute for industrially relevant enzymes.^28^

Existing approaches to intracellular peptide cyclization include use of transpeptidase^29^ and protein tags such as intein^30,31^ and SpyTag/SpyCatcher.^26^ However, long recognition sequences are needed for sortase A-mediated ligation (LPXTG)^29,32,33^ and the SpyTag/Catcher system (129 residues added).^26^ As recognition sequences remain in the modified protein, these methods introduce a traceable scar, which can alter the native protein fold. Ligation by intein splicing proceeds with a small ligation scar (C/S); however splice rates are slow (*k* = 3.7 × 10^–2^ s^–1^ for Npu intein^30^), with solubility issues reported in some intein-fused constructs.^34,35^ Consequently, additional methods for generating cyclic peptides are needed to address these limitations.

Here, we demonstrate that OaAEP1-C247A displays intracellular activity for peptide H2T cyclization in live *E. coli*, enabling *in situ* cyclization for direct production of a cyclic recombinant protein. Coexpression of OaAEP1-C247A and the murine dihydrofolate reductase (mDHFR) substrate resulted in a cyclic protein which can be isolated from the cell lysate. mDHFR was used as a proof-of-concept as the positions of the N- and C-termini are solvent exposed and in close proximity and therefore are well-suited for ligation (14.80 Å, Cα–Cα, PDB: 3D80). Moreover, the analogous DHFR from *E. coli* was previously employed as an exemplar for cyclization by split-intein and a cyanocysteine-mediated chemical cyclization method.^27,36^

## Results

### Polycistronic Gene Construct for Recombinant Cyclic Protein Production

**Figure 1:**
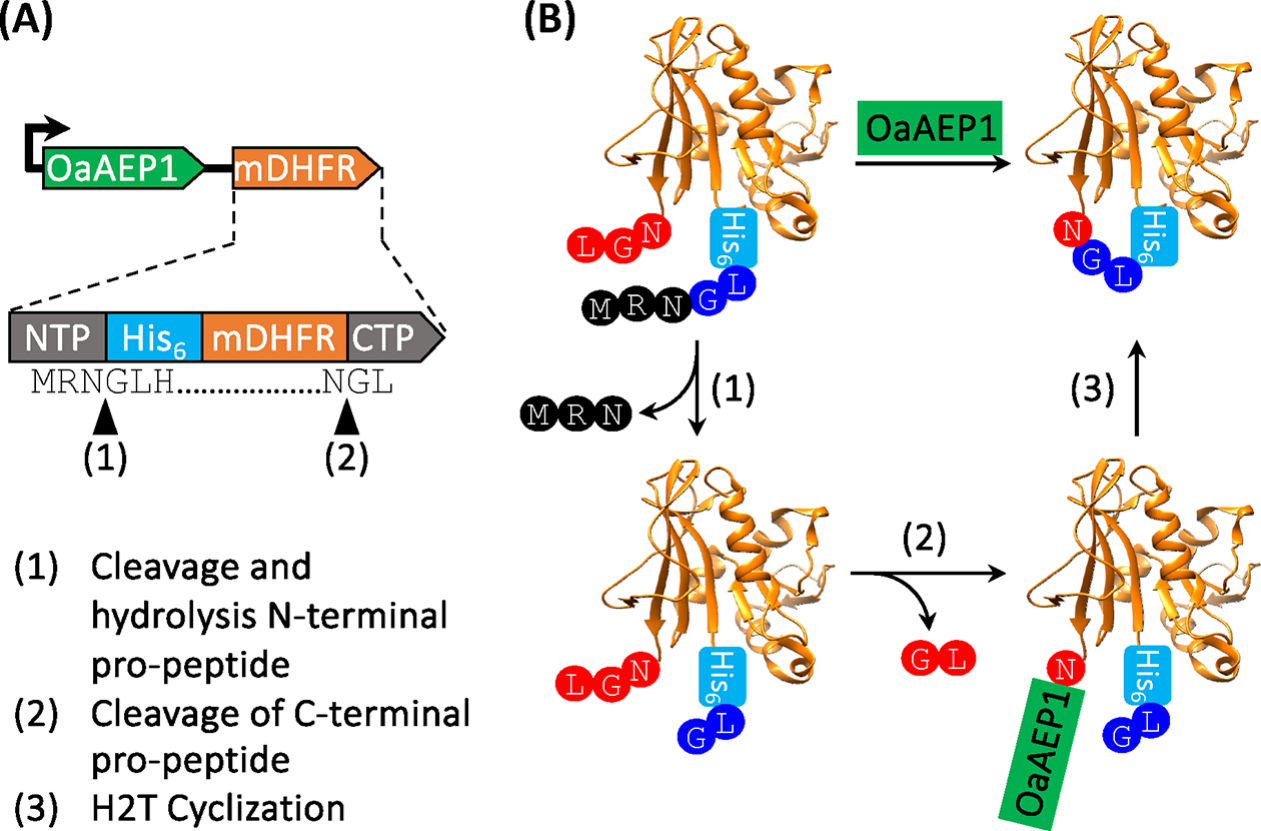
Direct production of cyclic mDHFR by recombinant gene expression. (A) Schematic coexpression system for generating cyclic mDHFR. The translated linear protein precursor to cyclic DHFR contains N- and C-terminal pro-peptides sequences (NTP and CTP, respectively) which are processed by OaAEP1 at the site indicated by black triangles. (B) Scheme of the proposed mechanism for mDHFR cyclization catalyzed by OaAEP1.

The genes encoding the enzyme OaAEP1-C247A and substrate mDHFR were subcloned into a pACYC-Duet coexpression plasmid for IPTG-inducible recombinant gene expression (Figure 1A, Figure S1). Specifically, the enzyme gene construct encodes the core domain of OaAEP1-C247A (residues 24–328) fused with an N-terminal His_6_-Ubiquitin tag (Figure S2). This construct was previously reported to generate soluble enzyme which was isolated for *in vitro* applications.^7^ The substrate gene encodes mDHFR with the addition of the OaAEP1 recognition sequences at the N- and C-termini as well as a His_6_ tag to enable purification (Figure 1A, Figure S1).

AEPs are known to exhibit protease and ligase activities.^7,37,38^ The protease activity of OaAEP1-C247A was utilized to remove the N-terminal peptide sequence from the linear mDHFR precursor, exposing an N-terminal Gly-Leu sequence for cyclization in a subsequent OaAEP1-C247A catalyzed reaction (Figure 1B, step 1). Although methionine aminopeptidase may also achieve a similar result in exposing the required Gly-Leu sequence for cyclization, relative expression levels and therefore the rate of Met removal may become a bottleneck for cyclization efficiency. Consequently, we chose to employ OaAEP1-C247A which is overexpressed and therefore likely to be more effective to process the mDHFR substrate. Finally, the C-terminal peptide is also processed by OaAEP1-C247A (Figure 1B, step 2) to form an enzyme–substrate intermediate, which is resolved by a nucleophilic substitution by the protein N-terminus to yield the H2T cyclic mDHFR (Figure 1B, step 3).

### Cyclic mDHFR Preparation and Characterization

**Figure 2:**
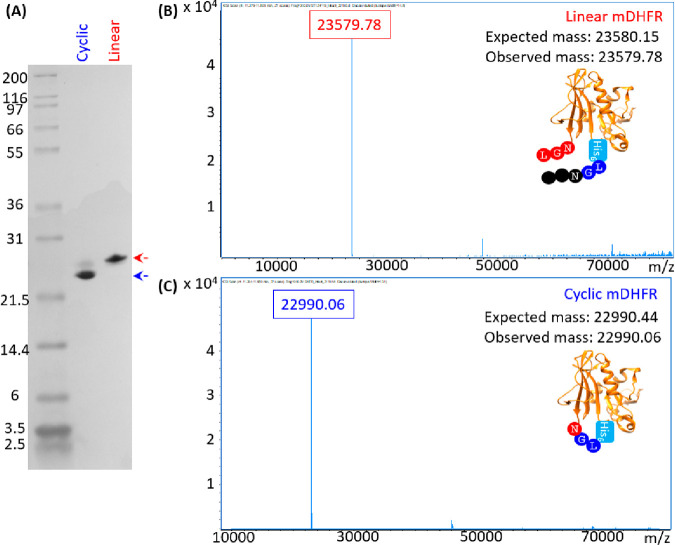
Mass analysis of cyclic and linear mDHFR. (A) SDS PAGE analysis of purified cyclic and linear mDHFR. (B) and (C) deconvoluted mass spectra of the purified cyclic and linear mDHFR demonstrates that upon cyclization 590 Da is lost due to loss of MRN from the N-terminus, GL from the C-terminus, and a water molecule during the condensation of N- and C-termini to form a native peptide bond.

mDHFR production and cyclization were detected by SDS-PAGE and mass spectrometry (MS) analyses. A 2-step purification by immobilized nickel affinity chromatography and size exclusion chromatography was used to isolate the linear mDHFR control, which produced a single prominent band by SDS-PAGE analysis (Figure 2A, red arrow). The calculated mass of the linear protein was detected by mass spectrometry analysis (Figure 2B). In contrast, when OaAEP1-C247A and mDHFR were coexpressed and purified using the same protocol, the major band during SDS-PAGE analysis migrated further (Figure 2A, blue arrow) corresponding to an increased retention time during size exclusion chromatography (SEC) (Figure S5), suggesting processing and cyclization by OaAEP1. Downward band shift in SDS PAGE analysis and increase SEC retention time can be attributed to (i) the more constrained cyclic topology of the protein and (ii) the loss of five amino acid residues and one water molecule, resulting in a loss of 590 Da during the OaAEP1-driven cyclization of mDHFR (three from the N-terminus (MRN), two from the C-terminus (GL), and H_2_O due to the formation of a native peptide bond). The faint secondary band observed in the cyclic DHFR sample does not align with the unprocessed linear protein and therefore may be due to the proteolytic processing of mDHFR by OaAEP1-C247A without cyclization or incomplete denaturation in SDS PAGE conditions. Nevertheless, mass spectrometry analysis detected only the desired cyclic protein but not the unprocessed or cleaved linear mDHFR masses (Figure 2C). The H2T cyclic topology was further validated by reaction with 2-phenylcarboxyaldehyde (2-PCA), a chemical labeling reagent which exclusively modifies the protein N-terminus.^39^ The linear mDHFR protein was quantitatively modified by 2-PCA, resulting in a molecular weight gain detected by MALDI-TOF MS analysis (Figure S6). In contrast, no mass change was detected when cyclic mDHFR was treated under the same reaction conditions (Figure S6), offering evidence for H2T cyclization. The protein yields from 1 L lysogeny broth cultures were comparable (3.5 mg for cyclic mDHFR vs. 3.2 mg linear mDHFR), highlighting the effectiveness of this novel process for cyclic protein production.

**Figure 3:**
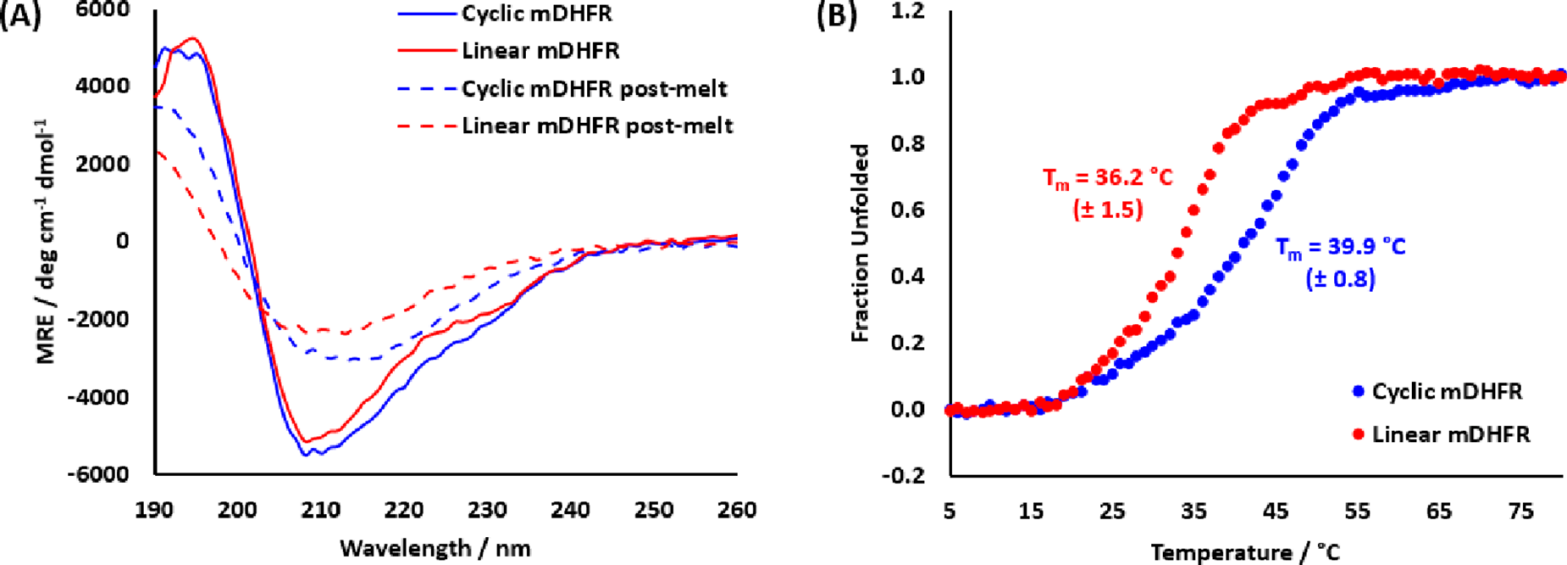
Circular dichroism analysis of cyclic and linear mDHFR (10 μM). (A) CD spectra (20 °C) of mDHFR before and after thermal denaturation. (B) Thermal denaturation profile by monitoring the MRE at 209 nm indicating an increase in *T*_m_ of 3.7 °C for the H2T cyclic protein. Fraction of unfolded protein was calculated using eq 1 in the Supporting Information. *T*_m_ was estimated by converting the data to MRE and fitting to the Boltzmann sigmoidal equation using OriginPro graphing and analysis software. *T*_m_ is reported here as the mean average of triplicate experiments with the standard error of the mean.

Circular dichroism (CD) studies were next performed to demonstrate that H2T cyclization did not significantly alter the global protein structure (Figure 3A). Both linear and cyclic mDHFR display strong negative mean residue ellipticity (MRE) at 209 nm and positive MRE signal at 195 nm which are consistent with the β-strand dominant content of the DHFR protein fold. Thermal denaturation analysis following the loss of the MRE signal at 209 nm displayed a thermal denaturation midpoint (*T*_m_) of 39.9 °C for cyclic mDHFR, an increase of 3.7 °C over the linear protein (36.2 °C) (Figure 3B). Neither protein was able to refold as demonstrated by the loss in signal for post-denaturation spectra.

### mDHFR Activity Assay

**Figure 4:**
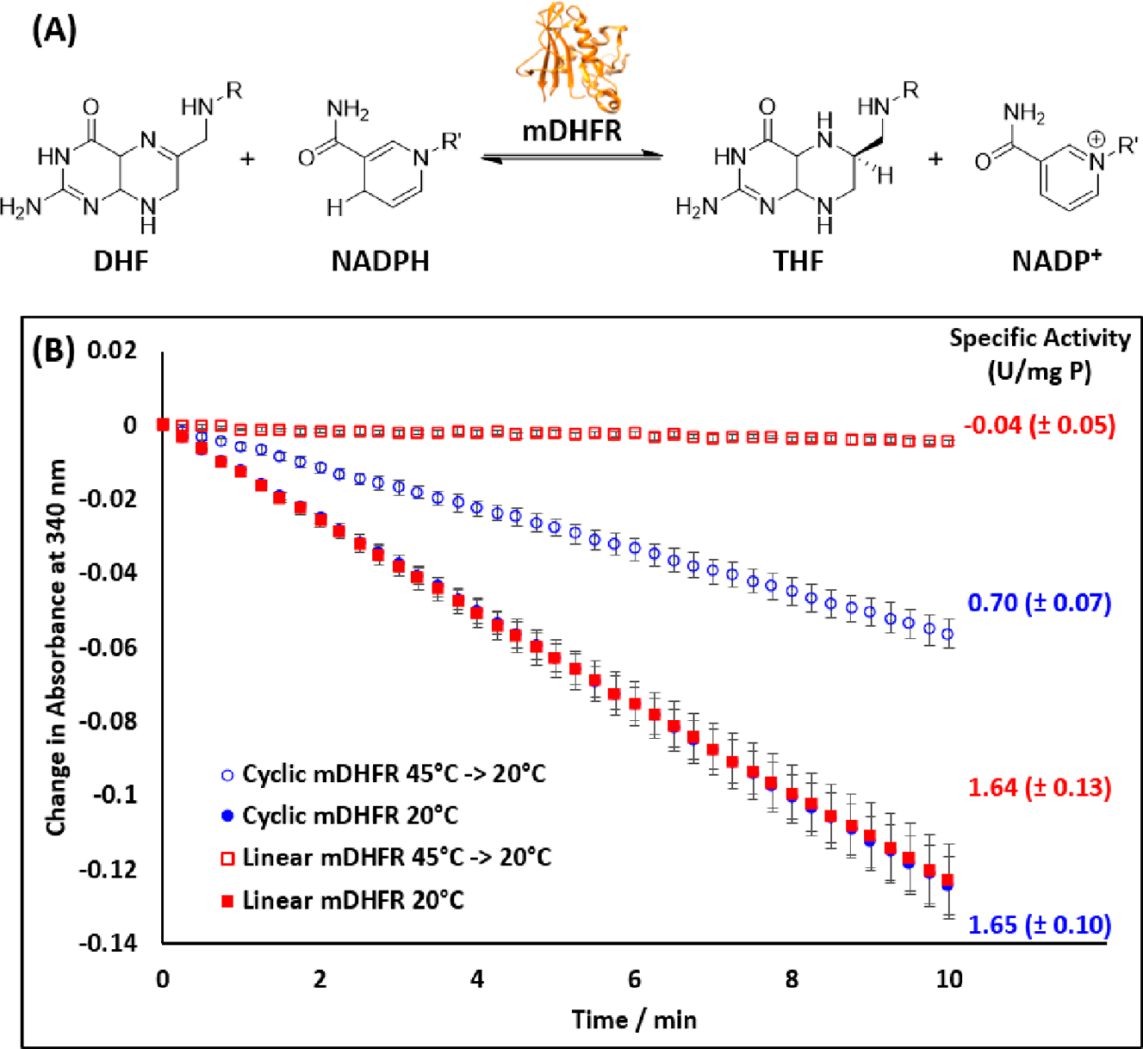
Activity assay for cyclic mDHFR following the change in absorbance at 340 nm by NADPH during mDHFR catalyzed reaction. (A) Schematic of the mDHFR catalyzed reduction of dihydrofolate (DHF) to tetrahydrofolate (THF) using NADPH as a cofactor. (B) UV/vis analysis monitoring the change in absorbance at 340 nm for turnover of NADPH at 20 °C (filled blue circles and red squares) showed comparable activity for both linear and cyclic versions. Following incubation at 45 °C for 15 min, the experiment was repeated at 20 °C and mDHFR activity again was monitored (open blue circles and red squares). Specific activity was calculated from the linear initial rate (first 2.5 min) using eq 2, Supporting Information. Data reported are averages with standard error of the mean from triplicate experiments.

The activity of cyclic mDHFR was next assayed by monitoring the reduction in absorbance at 340 nm due to the consumption of NADPH cofactor during the DHFR catalyzed reduction of dihydrofolate (DHF) into tetrahydrofolate (THF) (Figure 4A). No significant differences in specific activity were observed between the linear and cyclic protein at 20 °C (1.65 U mg^–1^, Figure 4B). To validate the apparent improved thermal stability observed in CD studies, mDHFR was incubated at 45 °C for 15 min, then cooled on ice before repeating the activity assay at 20 °C. Although 45 °C is above the observed *T*_m_ for cyclic mDHFR by CD analysis, the temperature was selected to achieve near-complete denaturation of the linear protein while retaining a proportion of folded cyclic protein (approximately 55% cyclic vs. 93% linear protein unfolded according to CD (Figure 3B)). Precipitation was observed in both cyclic and linear mDHFR upon incubation at 45 °C, which can be attributed to the loss of activity observed in the subsequent activity assay (Figure S7). In agreement with the CD thermal denaturation analysis, no reduction in absorbance at 340 nm was observed when linear mDHFR (preincubated at 45 °C) was used, indicating complete thermal denaturation of the enzyme (Figure 4B). On the other hand, NADPH consumption was detected when using the heat treated cyclic mDHFR. Lower specific activity was observed (0.70 U mg^–1^ for heat-treated vs. 1.65 U mg^–1^ for ambient; Figure 4B) proportional to the estimated fraction of unfolded protein (58% loss in activity vs. 55% of protein unfolded), and therefore can be attributed to the lower abundance of active enzyme due to partial denaturation of the cyclic protein.

## Discussion

H2T cyclization is a powerful protein engineering strategy to enhance thermal and chemical stability and proteolytic degradation resistance.^22,23,26,27^ The constrained topology can offer other benefits such as improved target binding affinity and bioavailability for therapeutic peptides.^21,22,24,25^ However, production of H2T cyclic peptides is challenging, often employing toxic and unsustainable reagents for chemical synthesis.^36,40^ Native chemical ligation offers a “greener” chemical approach which can proceed in aqueous conditions at ambient temperature, but strict requirements for denaturing conditions, N-terminal cysteines (or Cys surrogates), and C-terminal thioesters limit the versatility of this method.^41,42^ By contrast, biocatalytic approaches offer a much safer and more sustainable alternative but often require additional purification during recombinant preparation which is time and labor intensive. Here, we report a streamlined method to produce a H2T cyclic protein. Coexpression of the engineered transpeptidase OaAEP1-C247A with a substrate protein (mDHFR used here as a proof-of-concept) enables *in situ* cyclization during recombinant expression. Consequently, the cyclic product can be isolated from the host organism without having to purify each protein component individually. Cyclization was confirmed *in vitro* by SDS-PAGE and MS analysis (Figure 2). Loss of the protein N-terminus was further confirmed by reaction with 2-PCA, a site-specific chemical modifier of the protein N-terminus (Figure S6). Investigations by CD (Figure 3) and a mDHFR activity assay (Figure 4, Figure S4) demonstrated that H2T cyclization improved thermal stability (Δ*T*_m_ = 3.7 °C, Figure 3B) without affecting the protein fold or specific enzyme activity. DHFR activity assay following exposure to elevated temperature (45 °C) provided further evidence of improved thermal stability upon H2T cyclization. Heat-treated linear mDHFR showed no activity, while the cyclic mDHFR retained a level of activity proportionate to the estimated fraction of folded protein: 58% loss in activity vs. 55% of protein unfolded. To the best of our knowledge, this is the first intracellular application of AEP catalysis in a live recombinant organism outside of plant species.^43,44^ Recombinant *E. coli* systems offer fast growth rate, durability, ease of use, and low cost, and therefore *in situ* cyclization by OaAEP1-C247A in live *E. coli* represents a major step to improve access and usability of this powerful biocatalyst for protein modification. Notably, the linear control and cyclic protein were obtained in comparable yields (3.5 mg for cyclic mDHFR vs. 3.2 mg linear mDHFR), suggesting that our intracellular cyclization system was highly effective and that cyclic protein production in this study was limited by the abundance of substrate rather than enzyme activity. Cyclization of mDHFR was an ideal candidate for proof-of-concept due to the proximity of its N- and C-termini; the limits of this method for cyclic protein production should be tested with more challenging exemplars including intrinsically disordered proteins (IDPs) or smaller peptides. While OaAEP1 have been shown to cyclize the IDP merozoite surface protein 2 *in vitro*, reduced activity was observed.^14^ Therefore, the ability to cyclize IDPs may be a limitation and should be further explored.

Beyond the streamlined production of cyclic proteins, concomitant *in situ* peptide modification by OaAEP1-C247A offers a plethora of potential applications such as intracellular cyclic peptide library screening; the short and diverse substrate recognition of OaAEP1-C247A is ideal for generating cyclic peptide libraries.^7^ In addition, intracellular activity in *E. coli* would enable further engineering of the enzyme by directed evolution to enhance features such as activity, substrate recognition, protein stability, and solubility. Moreover, given that OaAEP1-C247A has been employed for site specific peptide and protein bioconjugation *in vitro*,^3−13^ intracellular applications can be explored to offer a convenient strategy for installing synthetic tags which cannot be genetically encoded (d-amino acids, isotopic labels, fluorophores, therapeutic warheads, etc.). In conclusion, we demonstrated a novel intracellular application of AEP catalysis for the direct production of recombinant cyclic proteins. Given the existing utility of AEPs *in vitro*, this intracellular method has the potential to become a broadly applicable tool for protein modification.

## Materials and Methods

Proteins (DNA and protein sequences in SI) were produced using standard recombinant expression methodologies and purified by various chromatography steps as detailed. mDHFR activity was measured by using a colorimetric assay kit (Sigma CD0340). A detailed description of the materials and methods utilized in this work is provided in the Supporting Information.

## Supporting Information Available

Detailed materials and methods; protein and gene sequences; plasmid maps; gel images further highlighting cyclic mDHFR production; activity data; SEC data; MS data and precipitation data (PDF)
