# Supplementary material for: Intracellular Application of an Asparaginyl Endopeptidase for Producing Recombinant Head-to-Tail Cyclic Proteins
Source: JACS Au. 2023 Nov 20;3(12):3290–6. doi: 10.1021/jacsau.3c00591 (PMC10751764; doi:10.1021/jacsau.3c00591)
Supplement: Supplementary file 1 — au3c00591_si_001.pdf [file au3c00591_si_001.pdf]

Supporting Information

Intracellular application of an asparaginyl endopeptidase for producing recombinant head-to-tail cyclic proteins

T. M. Simon Tang,[a] Jody M. Mason*[a]

* Corresponding author

[a] Department of Life Sciences, University of Bath, Claverton Down, Bath, North Somerset, BA2 7AY, UK

E-mail: j.mason@bath.ac.uk

Tel: +44 (0)1225386867

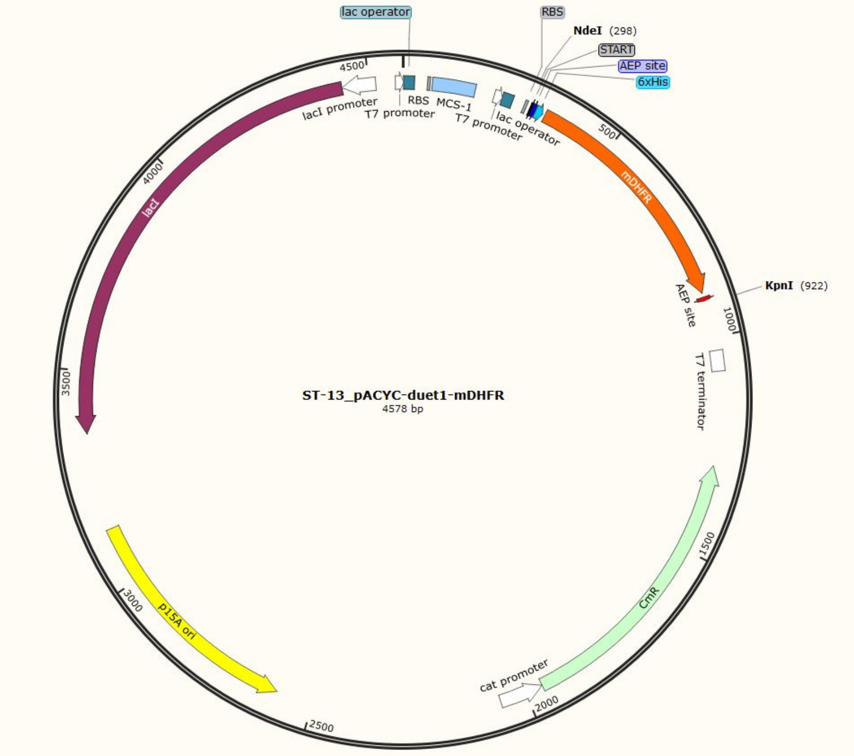

DNA sequence encoding for mDHFR:

ATGAGGAATGGATTACACCATCACCATCACCATGCTAGCGTTCGACCATTGAACTGCATCGTCGCCGTGTCCC
AAAATATGGGGATTGGCAAGAACGGAGACCTACCCTGGCCTCCGCTCAGGAACGAGTTCAAGTACTTCCAAAG
AATGACCACAACCTCTTCAGTGGAAGGTAAACAGAATCTGGTGATTATGGGTAGGAAAACCTGGTTCTCCATT
CCTGAGAAGAATCGACCTTTAAAGGACAGAATTAATATAGTTCTCAGTAGAGAACTCAAAGAACCACCACGAG
GAGCTCATTTTCTTGCCAAAAGTTTGGATGATGCCTTAAGACTTATTGAACAACCGGAATTGGCCAGTAAAGT
AGACATGGTTTGGGCAGTCGGAGGCAGTTCTGTTTACCAGGAAGCCATGAATCAACCAGGCCACCTCAGACTC
TTTGTGACAAGGATCATGCAGGAATTTGAAAGTGACACGTTTTTCCCAGAAATTGATTTGGGGAAATATAAAC
TTCTCCCAGAATACCCAGGCGTCCTCTCTGAGGTCCAGGAGGAAAAAGGCATCAAGTATAAGTTTGAAGTCTA
CGAGAAGAAAGACCCAGGCAGGAATGGATTATGA

Amino acid sequence encoding for mDHFR:

MRNGLHHHHHHASVRPLNCIVAVSQNMGIGKNGDLPWPPLRNEFKYFQRMTTTSSVEGKQNLVIMGRKTWFSI
PEKNRPLKDRINIVLSRELKEPPRGAHFLAKSLDDALRLIEQPELASKVDMVWAVGGSSVYQEAMNQPGHLRL
FVTRIMQEFESDTFFPEIDLGKYKLLPEYPGVLSEVQEEKGIKYKFEVYEKKDPGRNGL

Figure S1. *Plasmid map, DNA and amino acid sequences of recombinant mDHFR substrate. The protein (amino acid sequence in orange) was appended with a short linker, His$_6$ tag (in blue) for purification and AEP recognition sites at the N and C termini (black, underlined). NGL sequence near the N-terminus enables OaAEP1 to remove the upstream amino acids, revealing the cognate N-terminal recognition sequence (GL) for OaAEP1-mediated cyclisation.*

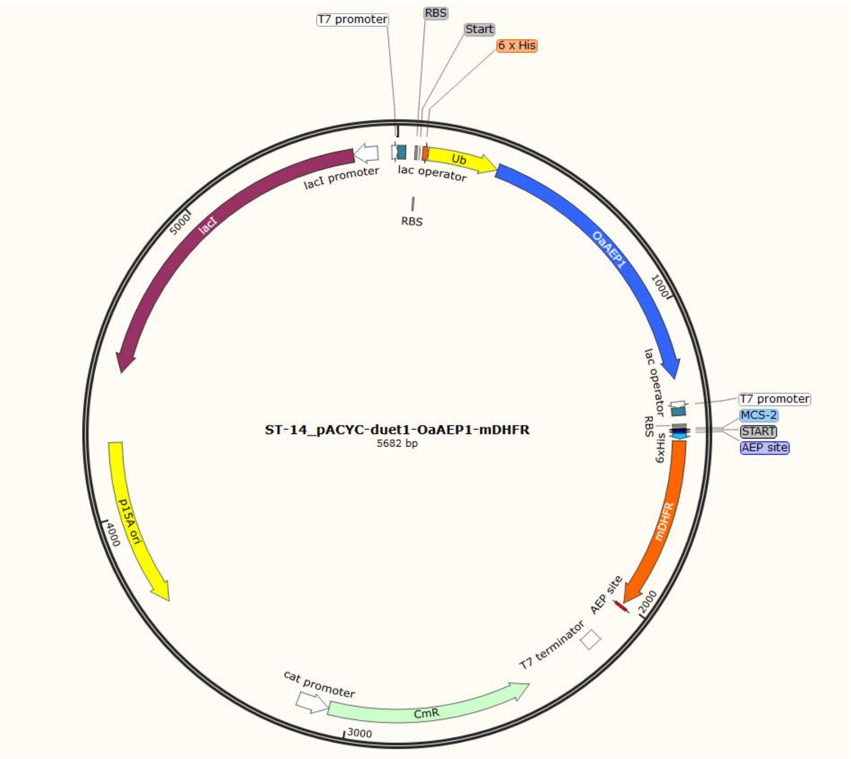

DNA sequence encoding for OaAEP1-C247A:

ATGGGCATGGCGCACCACCACCACCACCACATGCAGATCTTCGTTAAAACCCTGACCGGCAAGACCATTACCC
TGGAAGTGGAACCGAGCGACACCATCGAGAACGTGAAAGCGAAGATCCAAGACAAAGAAGGTATTCCGCCGGA
TCAGCAACGTCTGATTTTTGCGGGCAAGCAGCTGGAGGACGGTCGTACCCTGAGCGATTACAACATCCAAAAA
GAAAGCACCCTGCATCTGGTGCTGCGTCTGCGTGGTGGCGCGCGTGACGGTGATTATCTGCACCTGCCGAGCG
AGGTGTCTCGTTTCTTTCGTCCGCAGGAGACCAACGACGATCACGGCGAAGACAGCGTGGGTACCCGTTGGGC
GGTTCTGATTGCGGGTAGCAAGGGCTACGCGAACTATCGTCATCAGGCGGGCGTGTGCCATGCGTACCAAATC
CTGAAACGTGGTGGCCTGAAGGATGAGAACATCGTGGTTTTCATGTACGACGATATTGCGTATAACGAAAGCA
ACCCGCGTCCGGGTGTTATCATTAACAGCCCGCACGGCAGCGATGTGTATGCGGGTGTTCCGAAAGATTATAC
CGGCGAGGAAGTGAACGCGAAGAACTTCCTGGCGGCGATCCTGGGTAACAAAAGCGCGATTACCGGTGGCAGC
GGCAAGGTGGTTGACAGCGGTCCGAACGATCACATCTTTATTTACTATACCGACCACGGTGCGGCGGGCGTTA
TCGGTATGCCGAGCAAGCCGTACCTGTATGCGGACGAGCTGAACGATGCGCTGAAGAAAAAGCACGCGAGCGG
TACCTACAAAAGCCTGGTGTTCTATCTGGAGGCGTGCGAAAGCGGCAGCATGTTTGAGGGTATCCTGCCGGAA
GATCTGAACATTTACGCGCTGACCAGCACCAACACCACCGAAAGCAGCTGGGCCTACTATTGCCCGGCGCAGG
AGAACCCGCCGCCGCCGGAATATAACGTGTGCCTGGGCGACCTGTTCAGCGTTGCGTGGCTGGAGGACAGCGA
TGTGCAGAACAGCTGGTACGAAACCCTGAACCAGCAATATCACCACGTTGATAAGCGTATTAGCCACGCGAGC
CACGCGACCCAATACGGTAACCTGAAACTGGGCGAGGAAGGTCTGTTTGTGTACATGGGCAGCAACCCGGCGA
ACGACTAA

Amino acid sequence encoding for OaAEP1-C247A:

MGMAHHHHHHMQIFVKTLTGKTITLEVEPSDTIENVKAKIQDKEGIPPDQQRLIFAGKQLEDGRTLSDYNIQK
ESTLHLVLRLRGGARDGDYLHLPSEVSRFFRPQETNDDHGEDSVGTRWAVLIAGSKGYANYRHQAGVCHAYQI
LKRGGLKDENIVVFMYDDIAYNESNPRPGVIINSPHGSDVYAGVPKDYTGEEVNAKNFLAAILGNKSAITGGS
GKVVDSGPNDHIFIYYTDHGAAGVIGMPSKPYLYADELNDALKKKHASGTYKSLVFYLEACESGSMFEGILPE
DLNIYALTSTNTTESSWAYYCPAQENPPPPEYNVCLGDLFSVAWLEDSDVQNSWYETLNQQYHHVDKRISHAS
HATQYGNLKLGEEGLFVYMGSNPAND

Figure S2. *Plasmid map, DNA and amino acid sequences of recombinant OaAEP1-C247A employed for the production of cyclic mDHFR. Residues 24-328 of OaAEP1 (black, underlined) was employed with a C247A mutation. The enzyme was appended with a His$_6$ tag (in blue) to enable purification and a ubiquitin fusion (in orange) to enhance solubility.*

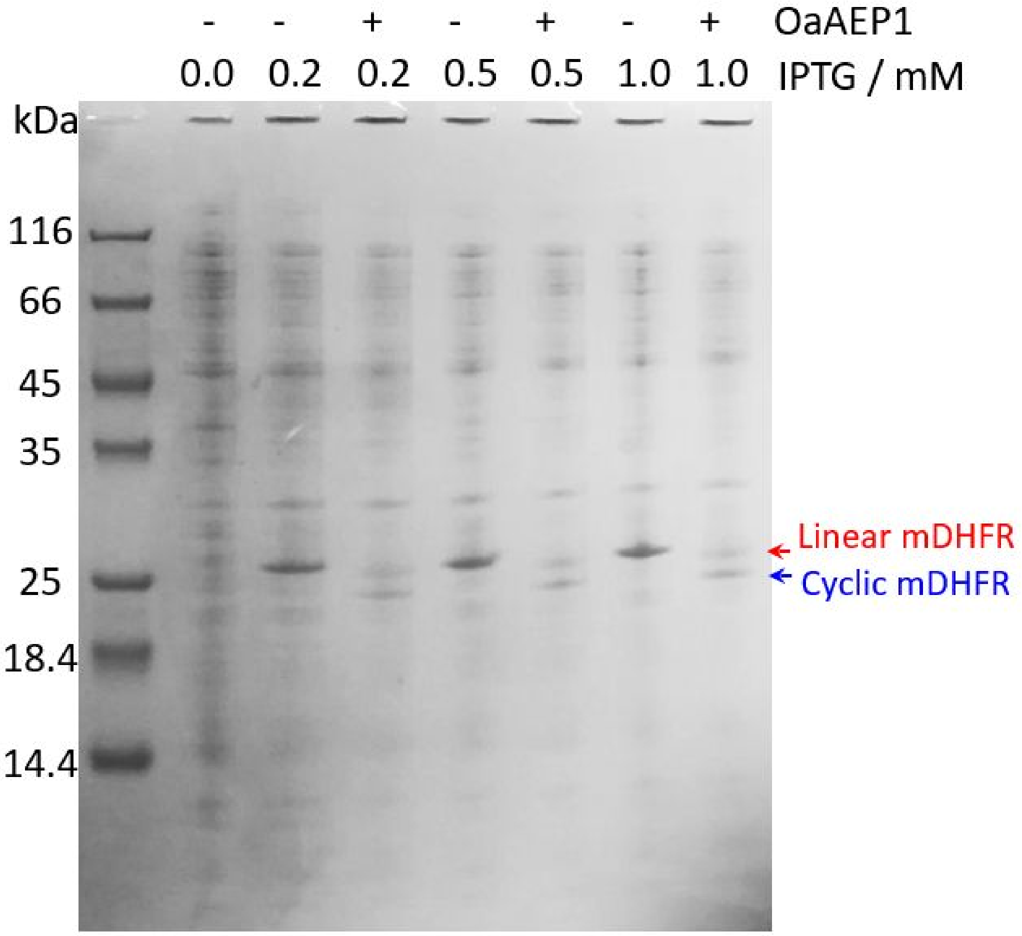

Figure S3. *SDS PAGE analysis of the protein contents in the host cell upon IPTG-induced gene expression using different concentrations of IPTG (0.2, 0.5, 1.0 mM final concentration).*

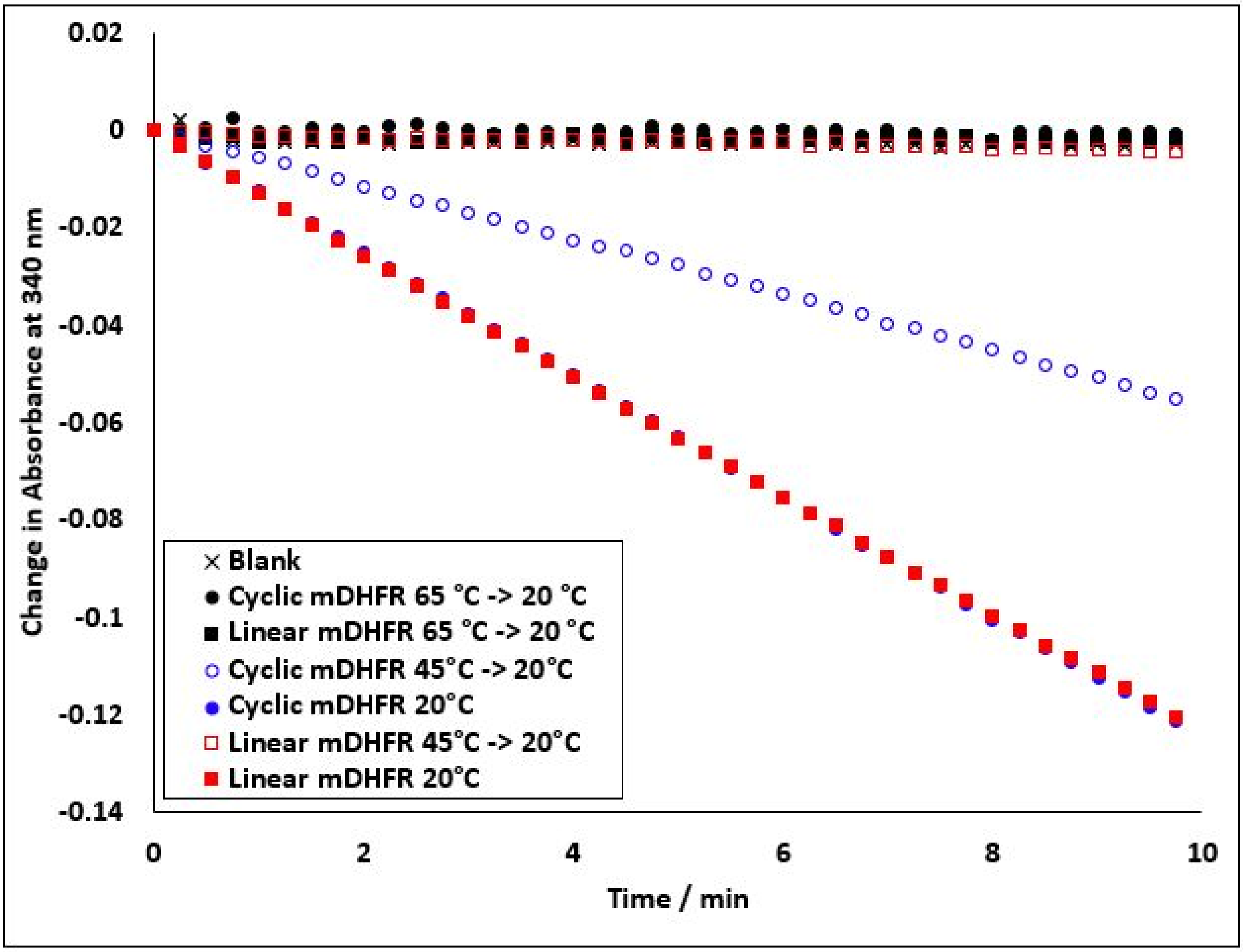

Figure S4. *UV/Vis analysis monitoring the change in absorbance at 340 nm for turnover of NADPH at 20 °C. Linear (squares) and cyclic (circles) mDHFR were incubated at 20 °C, 45 °C, or 65 °C before use in the assay (performed at 20 °C). A negative control of DHF and NADPH with no enzyme was employed as a blank (black crosses).*

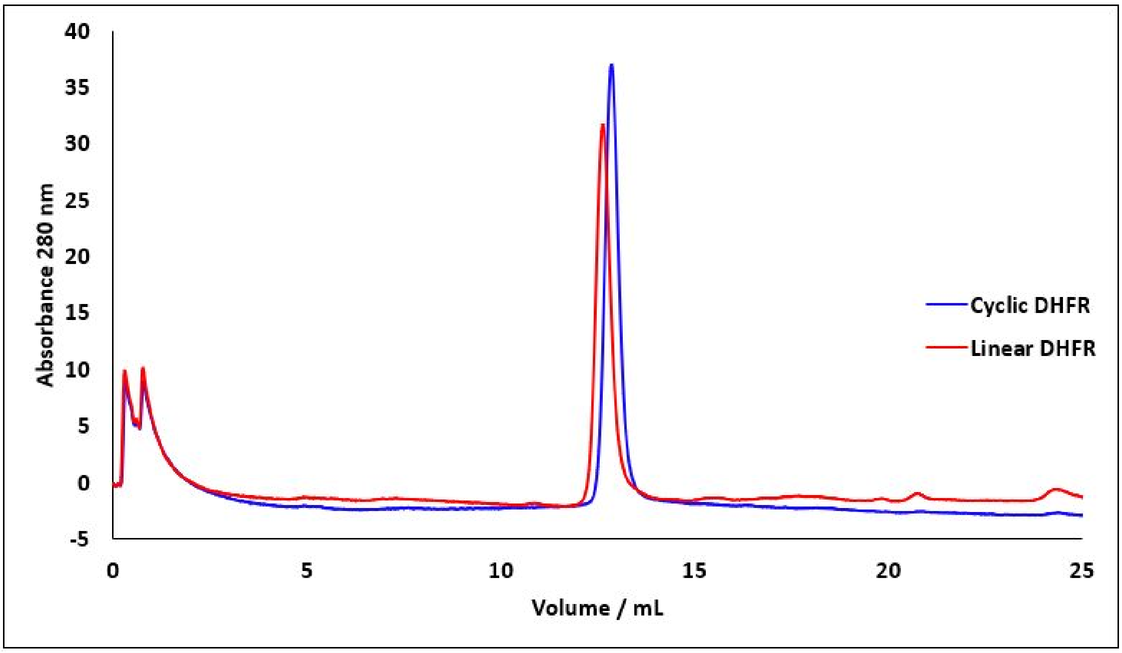

Figure S5. *Analytical size exclusion chromatography of linear (red) and cyclic (blue) mDHFR.*

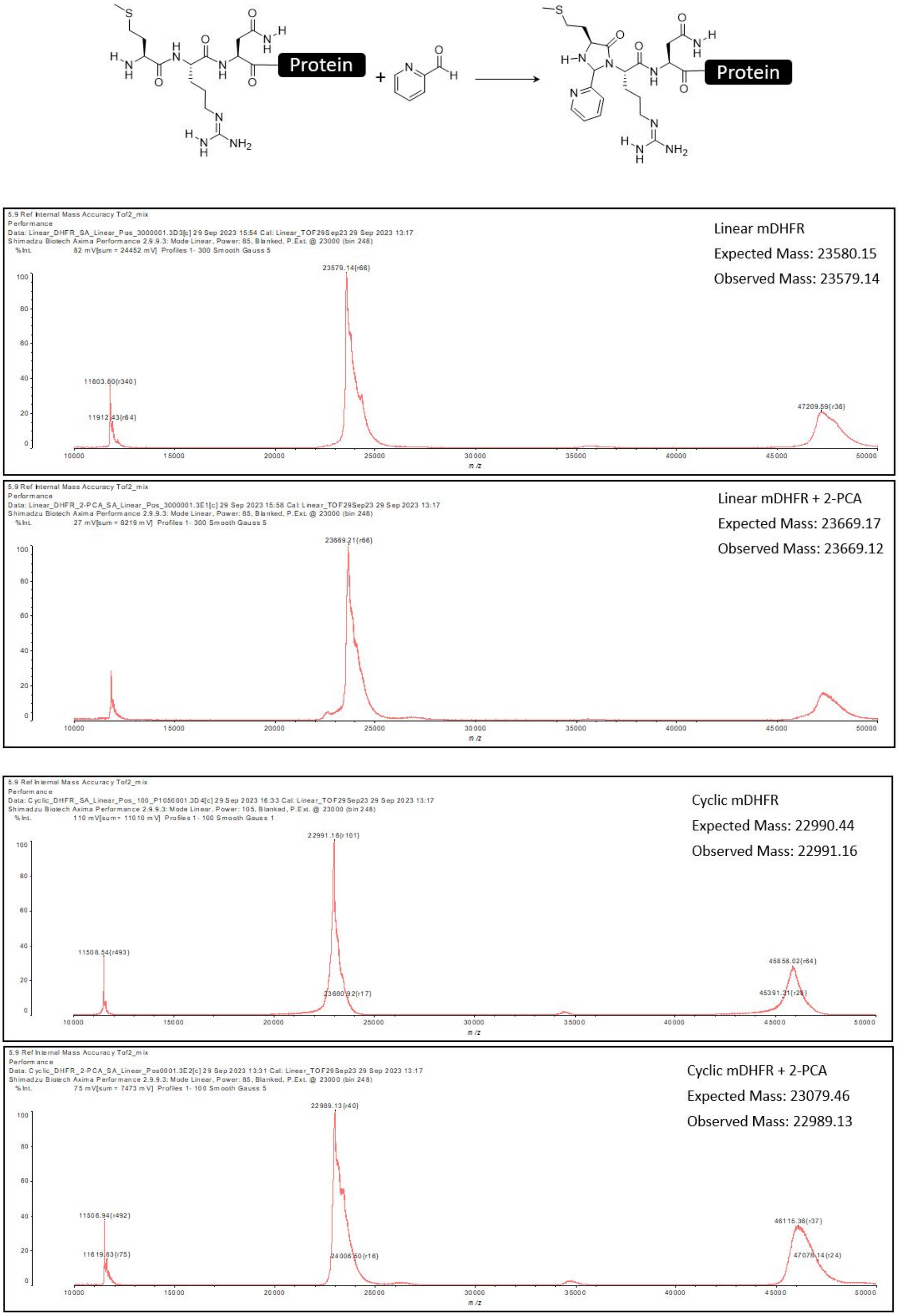

Figure S6. *Reaction scheme showing protein N-terminus modification by 2-PCA and MALDI-TOF MS of linear and cyclic mDHFR after overnight incubation with and without 2-PCA.*

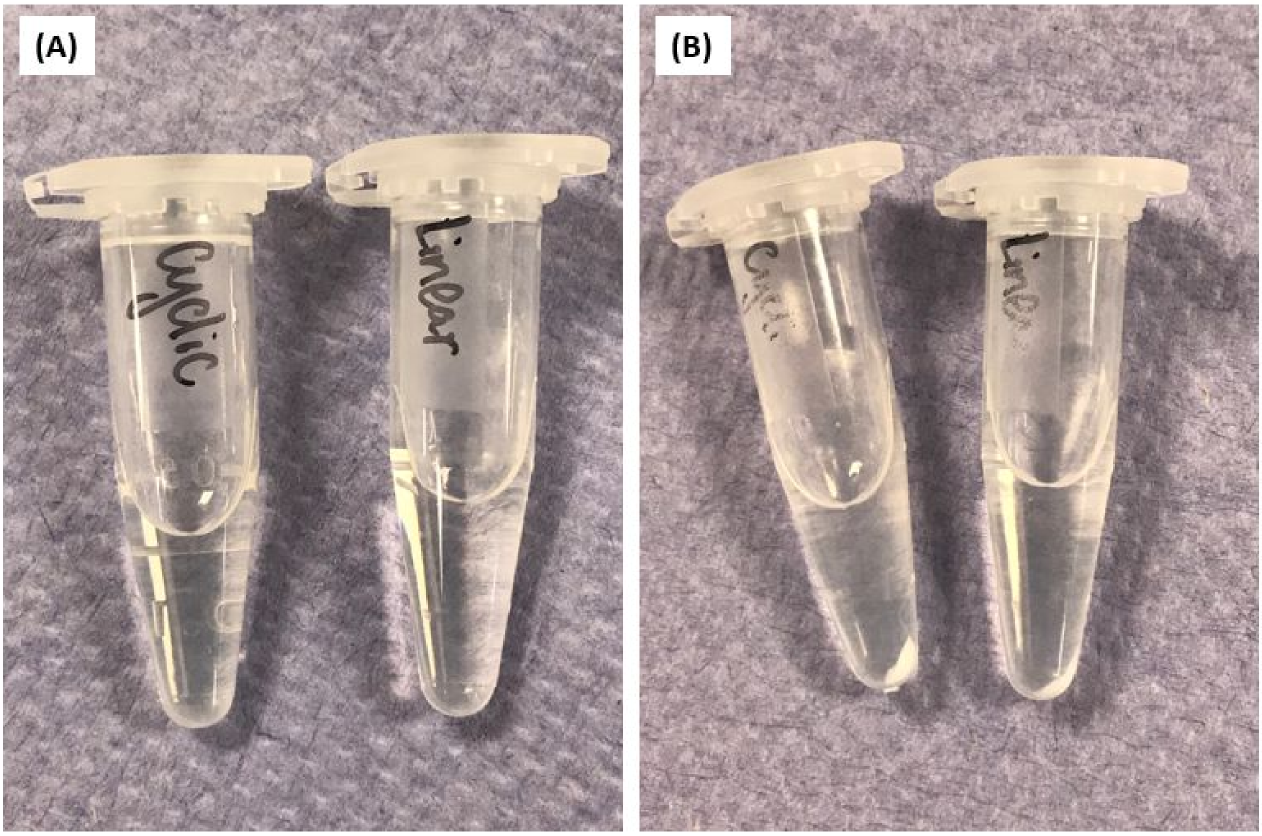

Figure S7. *(**A**) Linear and cyclic mDHFR remain stable and in solution at room temperature (20 °C, 15 min). (**B**) Protein precipitation was observed upon heat treatment (45 °C, 15 min) of both linear and cyclic mDHFR. However, while activity remains for cyclic mDHFR in solution after heat treatment, all activity for the linear counterpart is lost (Fig 4).*

Materials and Methods

General Information

Custom oligonucleotides and the pACYC-duet1 plasmid vector were purchased from Merck Sigma Aldrich. All restriction enzymes were purchased from ThermoFisher Scientific. Electrocompetent *E. coli*, buffers for molecular cloning, nucleotide triphosphate, Q5® High-Fidelity DNA polymerase, and NEBuilder® HiFi DNA assembly master mix was purchased from New England Biolabs. DNA Sanger sequencing performed by Eurofins Genomics.

Molecular Cloning

Linear mDHFR – The plasmid pACYC-Duet1-mDHFR was used for preparing linear mDHFR by IPTG induction. It was constructed by amplifying the genes encoding for His_6-mDHFR then subcloned between KpnI and NdeI restriction sites. The ligated product was transformed into *E. coli* strain DH5α. Sequences of all plasmids were confirmed by DNA Sanger sequencing.

Cyclic mDHFR – The plasmid pACYC-Duet1-OaAEP1-mDHFR was used for co-expression of OaAEP1-C247A and mDHFR by IPTG induction to prepare cyclic mDHFR. It was constructed by amplifying the gene encoding His_6-Ubquitin-OaAEP1-C247A by PCR using primers ST14I-F and ST14I-R into the plasmid pACYC-Duet1-mDHFR linearized by PCR using primers ST14V-F and ST14V-R. The PCR products were digested by DpnI (ThermoFisher) then assembled by Gibson Assembly using NEBuilder® HiFi DNA assembly master mix and transformed into *E. coli* strain DH5α. Sequences of plasmids were confirmed by DNA Sanger sequencing.

Primer ST14I-F
GGAGATATACCATGGGCATGGCG
Primer ST14I-R
CGGCCGCAAGCTTTTAGTCGTTCGCC
Primer ST14V-F
AAGCTTGCGGCCGCATAATG
Primer ST14V-R
GCCCATGGTATATCTCCTTATTAAAG

Recombinant expression and purification – Recombinant expression was performed in *E. coli* strain SHuffle T7 express. The cells were grown at 30 °C in LB medium until the OD_{600} reached 0.7, at which point protein production was induced by adding IPTG (to a final concentration of 0.2, 0.5 or 1.0 mM). After culturing at 16 °C for 18 h, cells were harvested by centrifugation at 4000 g and 4 °C for 20 min. Cell pellets were stored at -20 °C until purification. The cell pellet from a 1 L culture was suspended in 40 mL of lysis buffer containing 25 mM Tris.HCl (pH 7.5), 500 mM NaCl, 20 mM Imidazole, 0.1 mg/mL lysozyme, DNase I and cOmpete protease inhibitor cocktail. After lysis by sonication and clearance by centrifugation (14000g, 4 °C, 15 min), the supernatant was loaded onto a 5 mL of HisTrap FF (Cytiva). The column was washed with 10 column volumes of lysis buffer, and the remaining bound protein was eluted over a linear gradient from 20 to 500 mM imidazole over 20 column volumes. Fractions containing the desired protein were combined and concentrated using an Amicon Ultra-15 centrifugal filter unit (10 kDa MWCO) (Merck Millipore), then further purified by size exclusion chromatography (Superdex 75 pg 16/600, GE Healthcare) that had been pre-equilibrated in 25 mM Tris.HCl (pH 7.5), 100 mM NaCl, 1 mM DTT. Fractions containing desired protein were combined, concentrated using an Amicon Ultra-15 centrifugal filter unit (10 kDa MWCO) (Merck Millipore) then stored at -80 °C.

Mass Spectrometry (LC-MS) – LC-MS analyses were performed using an Agilent QTOF 6545 with Jetstream ESI spray source coupled to an Agilent 1260 Infinity II Quat pump HPLC with 1260 autosampler, column oven compartment and variable wavelength detector (VWD). The MS was operated in positive ionization mode (gas temperature at 350°C, drying gas flow at 11 L min^{-1}, nebulizer gas flow at 50 psi, sheath gas temperature at 400°C, sheath gas flow at 12 L min^{-1}). The VCap, Nozzle, Fragmentor and Skimmer voltages were set to

4000, 1000, 200 and 65 V respectively. The chromatographic separation (2 µL sample injection) was performed on an Agilent polymeric reversed phase (PLRP-S, 2.1 x 50 mm, 3 µm, 300 Å) at 0.2 mL/min using mobile phase A (H_2O with 0.1% formic acid), and B (acetonitrile with 0.1% formic acid). Gradient elution started at 5% B, changed to 50% B at 8 min, then to 90% B at 9 min, held at 90% B until 10 min, then returned to 5% B for re-equilibration in a total 13 min run. Data analyses were performed in MassHunter BioConfirm 10.0.

Analytical Size Exclusion Chromatography – Analytical size exclusion chromatography was performed by injecting 100 µL of protein sample on a Superdex 75 Increase 10/300 GL (GE Healthcare) with 25 mM Tris-HCl (pH 7.5), 100 mM NaCl flowing at 0.8 mL min^{-1} on an AKTA FPLC system. Elution and therefore retention time was monitored by measuring the absorbance at 280 nm wavelength.

Matrix Assisted Laser Desorption Ionisation Mass Spectrometry (MALDI-TOF MS) – To co-crystallise the mDHFR reaction mixture for MALDI-TOF analysis, a ground steel well plate was wetted with 0.5 µL matrix (Sinapic acid, 10 mg mL^{-1}), followed by 0.5 µL of reaction mixture, then 0.5 µL matrix. The sample was allowed to dry in ambient conditions before analysis. MALDI-TOF MS spectra were recorded on a Shimadzu Axima iD Plus Performance MALDI-TOF TOF spectrometer fitted with a 6mW laser operating at 337 nm recording in linear positive mode.

Chemical modification of protein N-terminus – 2-pyridinecarboxyaldehyde (2-PCA) was purchase from Merck. N-terminal protein modification was performed according to reported protocol.[1] Briefly, mDHFR (linear or cyclic) was incubated with 2-PCA in 25 mM sodium phosphate buffer (pH 7.5) at a final concentrate of 15 µM mDHFR and 2 mM 2-PCA. The reaction mixture was incubated at room temperature (20 °C) overnight (19 h). The reaction mixtures were analysed by MALDI-TOF MS

Circular Dichroism (CD) – CD measurements were taken on an Applied Photophysics Chirascan with 200 µL samples in 1 mm path-length CD cells. Protein samples were diluted in 20 mM potassium phosphate (pH 7.4), 100 mM potassium fluoride. For full spectra, three scans between 190 and 300 nm were collected with a bandwidth of 1 nm and data sampled at a rate of 0.5 s^{-1}. These scans were averaged, a blank was subtracted, and the values were converted to molar residue ellipticity (MRE). Thermal denaturation experiments were performed by measuring the ellipticity at 209 nm over a temperature gradient from 4 to 90°C at 1°C increments. Post-melt scans at 20°C confirmed the proteins were irreversibly denatured as they did not overlay with the pre-melt scans. To determine the melting temperature (T_m), the thermal denaturation curves were converted to MRE and fitted to the Boltzmann sigmoidal equation using OriginPro graphing and analysis software.
To estimate the fraction of unfolded protein during thermal denaturation, lower and upper bounds of each data set were linearly extrapolated using the 10 data points at 4 – 13°C and 76 – 85°C, respectively. The MRE from each sample was then fitted to respective lower and upper bounds using the following equation:

$$Fraction\ of\ unfolded\ protein = \frac{MRE - Lower\ bound}{Upper\ bound - Lower\ bound} \qquad (1)$$

The fraction of unfolded protein was plotted against temperature to present normalized thermal denaturation profiles of linear and cyclic mDHFR.

DHFR activity assay – A colorimetric assay kit (Merck CS0340) was used to measure the activity of purified mDHFR enzymes. Cyclic or Linear-mDHFR (50 nM) and NADPH (60 µM) were mixed in assay buffer. For the blank reaction, a volume of assay buffer was added instead of enzyme (**Fig S4**). Reactions were initiated by the addition of DHF (50 µM) and the absorbance at 340 nm of samples was measured using a 10 mm path-length quartz cuvette in a Varian Cary 50 UV-Vis spectrophotometer. The specific activity was calculated using the following equation:

$$Specific\ activity = \frac{(\Delta absorbance\ \mathrm{min}^{-1}{}_{\mathrm{sample}} - \Delta absorbance\ \mathrm{min}^{-1}{}_{\mathrm{blank}})}{12.3 \times \mathrm{mg\ protein}} \qquad (2)$$

To investigate thermal stability, mDHFR were incubated at 20 °C, 45 °C, or 65 °C, then cooled on ice, before use in the activity assay (**Fig 3, Fig S4**).

References:

(1) MacDonald, J. I.; Munch, H. K.; Moore, T.; Francis, M. B. One-step site-specific modification of native proteins with 2-pyridinecarboxyaldehydes. *Nature Chemical Biology* **2015,** *11*, 326.
